# Functional and Structural Diversity of Insect Glutathione S-transferases in Xenobiotic Adaptation

**DOI:** 10.7150/ijbs.77141

**Published:** 2022-09-11

**Authors:** Sonu Koirala B K, Timothy Moural, Fang Zhu

**Affiliations:** 1 Department of Entomology, Pennsylvania State University, University Park, PA 16802, USA.; 2 Huck Institutes of the Life Sciences, Pennsylvania State University, University Park, PA 16802, USA.

**Keywords:** enzyme, metabolic detoxification, host adaptation, oxidative stress, pesticide resistance

## Abstract

As a superfamily of multifunctional enzymes that is mainly associated with xenobiotic adaptation, glutathione S-transferases (GSTs) facilitate insects' survival under chemical stresses in their environment. GSTs confer xenobiotic adaptation through direct metabolism or sequestration of xenobiotics, and/or indirectly by providing protection against oxidative stress induced by xenobiotic exposure. In this article, a comprehensive overview of current understanding on the versatile functions of insect GSTs in detoxifying chemical compounds is presented. The diverse structures of different classes of insect GSTs, specifically the spatial localization and composition of their amino acid residues constituted in their active sites are also summarized. Recent availability of whole genome sequences of numerous insect species, accompanied by RNA interference, X-ray crystallography, enzyme kinetics and site-directed mutagenesis techniques have significantly enhanced our understanding of functional and structural diversity of insect GSTs.

## Introduction

Insects constitute the largest class of animals encompassing about 53% of all living species on our planet 1. Many of these species (about 45%) are herbivores by partly or completely feeding on plants and represent a significant proportion of pests or pollinators for economically important crops. Annually, the economic association of these herbivores with food production in the U.S. exceeds $50 billion 1, 2. The arms race between plants and insect herbivores have driven their coevolution for hundreds of millions of years. To defend against insect herbivores, plants produce a wide range of chemical compounds, such as terpenoids, alkaloids, anthocyanins, glucosinolates, phenols, quinones, plant protease inhibitors (PIs), and herbivore-induced plant volatiles (HIPVs). These chemicals either directly reduce herbivores fitness or indirectly attract herbivores' natural enemies and enhance the effectiveness of their natural enemies 3, 4. In response, herbivores have simultaneously developed countermeasures against plant defense compounds 5. Such adaptive capability has been proposed to be co-opted by herbivore arthropod pests for pesticide resistance when they are exposed to the pressure of recently introduced synthetic pesticides 6-8. The similarities in modes of action between various naturally occurring chemical substances released by plants and synthetic pesticides further supports the possible linkage between host plant adaptation and currently prevailed pesticide resistance 9. In fact, more than 50% of all agrochemicals are natural products or derived from natural products 10-12.

**Figure 1:**
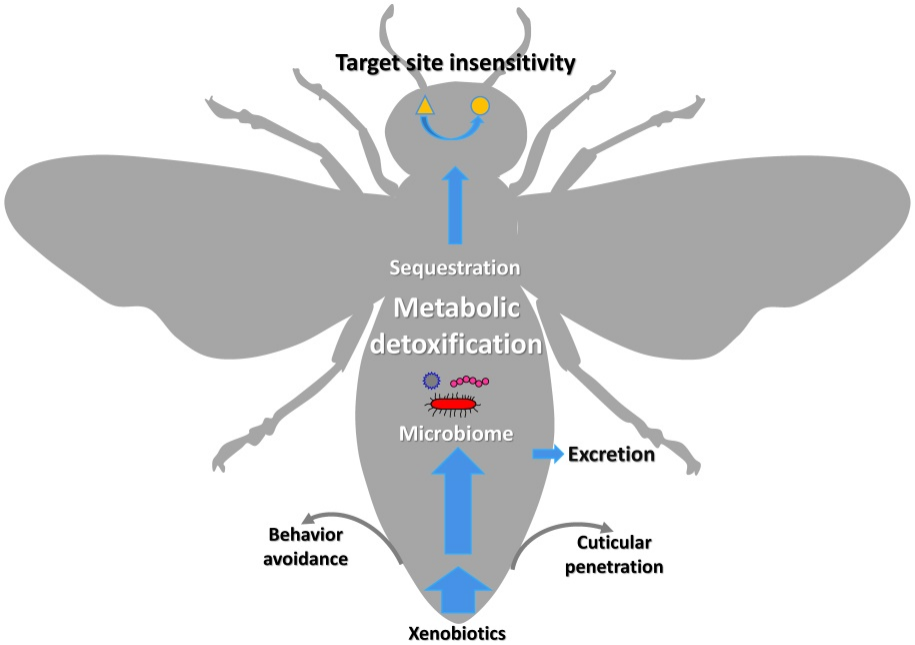
Graphic representation of the xenobiotic adaptations in arthropods that have evolved through different mechanisms**.** The thickness of the blue arrows represents the concentration of xenobiotics.

The xenobiotic adaptation in arthropods evolves through multiple mechanisms (Figure 1) 13, 14, including reduced penetration through the cuticle, behavioral avoidance 15, 16, microbiome-mediated detoxification 17-20, enhanced metabolic detoxification 21-25, enhanced sequestration or excretion 13, 19, 26, 27, and target site insensitivity 28-32. Among them, enhanced metabolic detoxification and target site insensitivity are the most common mechanisms 5, 33-35.

**Figure 2:**
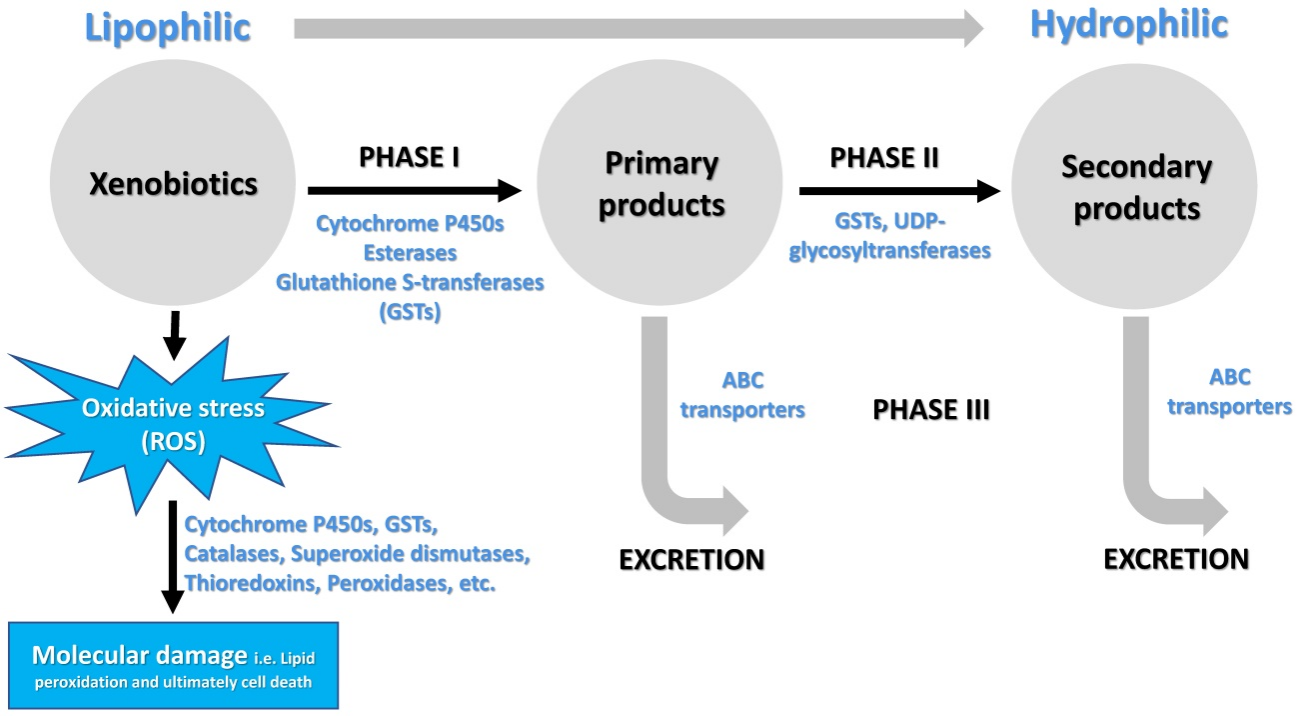
Schematic illustrating the process of xenobiotic metabolism, which encompasses three phases I, II, III (Adopted from 137) as well as xenobiotic induced oxidative stress and molecular damage.

There are several categories of enzymes involved in the metabolism of lipophilic xenobiotics and their conversions into less toxic compounds exhibiting increased hydrophilicity (Figure 2). The major enzyme superfamilies comprise cytochrome P450 monooxygenases (P450s), glutathione S-transferases (GSTs), carboxylesterases (COEs), ATP-binding cassette (ABC) transporters, and UDP-glycosyltransferases (UGTs) 5, 26, 36-39 (Figure 2). In general, three phases of metabolic detoxification of xenobiotics have been often described in the literature. Phase I detoxification includes oxidation, reduction, and hydrolysis of lipophilic substances carried out by a variety of enzymes. Phase II reactions involve conjugation of hydrophilic compounds (i.e. glutathione) to xenobiotics and/or phase I products to produce more hydrophilic products. In Phase III, products of phases I and/or II are excreted from cells by multidrug resistance proteins and other ABC transporters 37. Among metabolic detoxification enzymes, GST is a family of multifunctional enzymes that are ubiquitously present in eukaryotes and prokaryotes, playing an important role in the detoxification of numerous endogenous and exogenous compounds. As phase II enzymes, GSTs detoxify chemical compounds through catalyzing nucleophilic attack by the thiol group in reduced glutathione (GSH) on a wide range of electrophilic substrates 37, 40, 41. These substrates can be plant allelochemicals, pesticides, environmental pollutants, or byproducts of oxidative stress 40, 42. GSTs are also involved in the phase I detoxification process such as dehydrochlorination of 1,1,1-Trichloro-2,2-bis(*p*-chlorophenyl) ethane (DDT) to less toxic 1,l-Dichloro-2,2-bis(*p*-chlorophenyl) ethylene (DDE) 22, 43, 44. In addition, GSTs may participate in the passive non-catalytic binding of substrates and sequestration, which prevents the binding of xenobiotics to their target proteins 45-48.

Besides triggering a sequence of events that cause toxic outcomes, exposure to xenobiotics leads to induced oxidative stress, generating an over production of reactive oxygen species (ROS) 49 and consequently triggering oxidative damage to macromolecules such as proteins, lipids, and nucleic acids 50, 51. To cope with oxidative stress, arthropods evolve antioxidant enzymes for removing excess ROS to maintain intracellular redox homeostasis and avoid oxidative damage. These antioxidant enzymes include GSTs, catalases, superoxide dismutases, thioredoxins, glutathione peroxidases, glutaredoxins and thioredoxin peroxidases 52, 53 (Figure 2). Insect GSTs not only are involved in xenobiotic conjugation but also play roles in protection against oxidative stress caused by exposure to pesticides 46, plant allelochemicals 54, as well as various other abiotic factors 55, 56. Recent reviews had summarized functions of insect GSTs in insecticide resistance 22, 57. Therefore, the current review focuses on the structural and functional divergence of GST enzymes in arthropods and their potential roles in xenobiotic adaptation.

## Classification of GSTs

In eukaryotes and aerobic prokaryotes, GSTs are grouped into at least four major protein families: cytosolic GSTs, mitochondrial GSTs, microsomal GSTs, and bacterial Fosfomycin-resistance proteins 40, 42, 58-59. Mitochondrial GSTs are known as the kappa class in mammals and are mostly found in the mitochondrial matrix 60 and peroxisomes 61. Research has indicated that mitochondrial GSTs in humans play important roles in the detoxification of lipid peroxide and lipid metabolism 61. Microsomal GSTs belong to the MAPEG family (membrane‐associated proteins in eicosanoid and glutathione metabolism), which play a significant role in the reduction of lipid peroxidation and xenobiotic detoxification 62, 63. In contrast to mitochondrial and microsomal GSTs, cytosolic GSTs are present in the cytoplasm and are soluble 44. Both microsomal and cytosolic GSTs are found in arthropod species; however, the gene numbers in microsomal GSTs are fewer than the cytosolic GSTs (Table 1) 62, 64, 65. Moreover, cytosolic GSTs, which are typically 200-250 amino acids in length, form homo- or hetero-dimers, whereas microsomal GSTs are smaller (nearly 150 amino acids) and form trimers 62, 66. Arthropod cytosolic GSTs are classified into several classes according to the sequence similarities and structural properties: Delta, Epsilon, Omega, Sigma, Theta, Zeta, and unclassified classes (Table 1). Among these classes, Omega, Sigma, Theta, and Zeta classes are identified in most metazoans 67 and some aerobic prokaryotes 58, 68. Epsilon and Delta classes are insect-specific 62, 69. These two classes of cytosolic GSTs have undergone species-specific gene expansion to a great extent 41, 64, 65. It was hypothesized that such expansion might have occurred during adaptation to environmental selection pressure. This expansion or duplication of genes resulted in sequence variations that expanded substrate functionality and/or responses to environmental stresses 62, 70, 71.

Arthropod cytosolic GSTs are mainly involved in xenobiotic adaptation. With genomes of arthropod species available, gene number variation in each class of cytosolic GSTs has been observed in different species (Table 1). It has been hypothesized that a smaller number of cytosolic GST genes in the European honey bee (*Aphis mellifera*) than in other insect species might be associated with pesticide sensitivity and reduction in vitality 72. Besides, predator *Orius laevigatus*, monophagous or oligophagous agricultural pests *Nilaparvata lugens* and *Diaphorina citri* possess a low number of Delta, Epsilon, and total GSTs in their genomes (Table 1). The deficit in the number of GST genes is likely due to the low degree of exposure to xenobiotics in their natural environment.

## General structure of cytosolic GSTs

**Figure 3:**
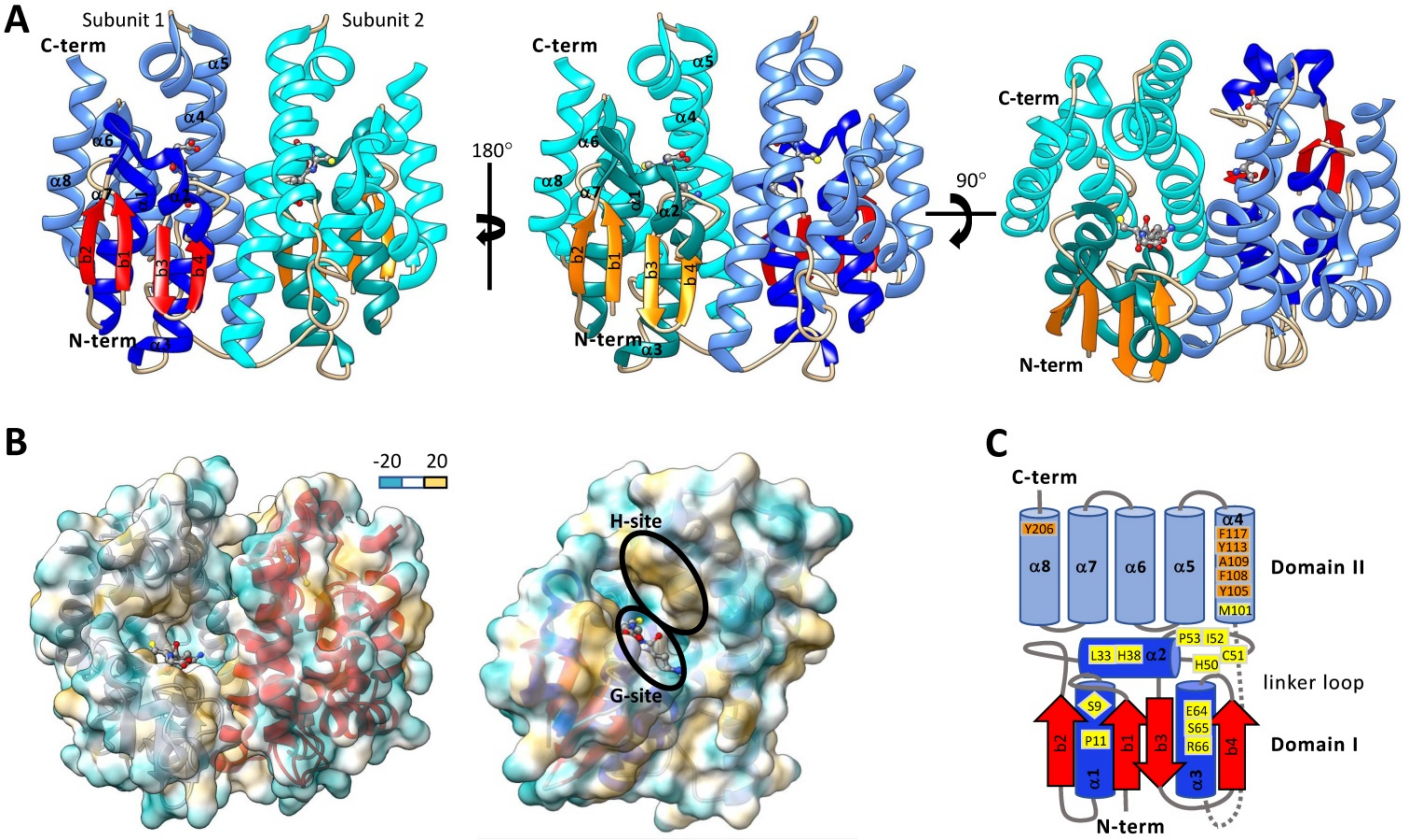
Structures of representative insect cytosolic GSTs. **A.** Ribbon diagram of *Drosophila melanogaster* dmGSTD1 (PDB: 3MAK). In subunit 1, the N-terminal domain I helices are shown in dark blue, and β-strands are shown in red, and the C-terminal domain II helices are shown in light blue. In subunit 2 the domain I helices are dark cyan β-strands are orange, and the domain II helices are light cyan. Glutathione is colored by the element and is shown in ball and stick format. **B.** Dimer (left) and monomer (right) ribbon diagrams of dmGSTD1 (PDB: 3MAK) overlayed with lipophilic surface representation. **C.** Secondary structure map of *Anopheles dirus* GSTD3-3 (PDB: 1JLV). Domain I helices are shown in dark blue and beta strands are shown in red. Domain II helices are shown in light blue. Loop regions for both domains I and II are shown in grey. The link region loop is dashed. Ribbon and surface diagrams were generated with UCSF ChimeraX.

Typically, cytosolic GSTs are hetero- or homo-dimeric proteins and are about 23-30 kDa per monomer. It has been proposed that heterodimer formation is restricted to both subunits being from the same class due to dimer interface compatibility interactions. Crystallographic evidence shows that homodimer subunits are related by a two-fold symmetry axis (Figure 3A&B) 73. Each monomer of a cytosolic GST is composed of an N-terminal domain (domain I) and C-terminal domain (domain II). N-terminal domain has β strands and α helices, and the C-terminal domain consists of helices 42, 62, 74. Domain I exhibits the structurally conserved thioredoxin-like fold motif βαβαββα (Figure 3A&C) 44, 68, 75. The N-terminal domain I is connected to the C-terminal domain II by a linker loop region consisting of around 10 amino acids 42, 62, 76. The C-term domain II consists of 4-8 helices depending on the GST class 42, 62, 73, 76. One of the striking features of GST is that each subunit has two ligand-binding sites - “G” site and “H” site (Figure 3), which together constitute the catalytic active site 62, 77. The G-site is more hydrophilic and exhibits a higher degree of sequence conservation within GST families than the H-site 42. The G-site is predominantly contained in the N-terminal domain and binds GSH and primes the thiol sulfur for nucleophilic attack on an electrophilic substrate 77-79. In contrast, the hydrophobic H-site is predominantly contained in the C-terminal domain adjacent to the G-site and binds electrophilic substrates 62, 80. The amino acid residues that make up the H-site are involved in recognizing and binding various exogenous and endogenous compounds and positioning their electrophilic centers for attack by the nucleophilic GSH.

### G-site

The type and position of amino acids in the active site of GSTs (G-site and H-site) play important roles in substrate binding affinity and catalytic function 74. It is thus important to make a comparison among different GSTs to understand their evolution and functions in the detoxification of diverse chemical substrates. With the aid of X-ray crystallography and site-directed mutagenesis techniques, the roles of GST active site amino acid residues were identified and evaluated 62, 81. In *Anopheles dirus*, a delta GST GSTD3-3 (PDB: 1JLV), G-site residues Ser-9, Pro-11, Leu-33, His-38, His-50, Cys-51, Ile-52, Pro-53, Glu-64, Ser-65, Arg-66, and Met-101 are within a 4.0 Å distance cutoff of GSH (Figure 3C) 82, 83. Among them, the Ser-65 residue was generally conserved across all GST classes. Ser-65 forms a hydrogen bond with the GSH γ-glutamyl carboxylate 80, 83. Additionally, Ile-52 and Glu-64 were generally maintained as either hydrophobic or polar residues across GST classes 82. The Ile-52 backbone amide forms a hydrogen bond with the backbone carbonyl of the GSH cysteinyl group and Glu-64 forms a salt-bridge with the amino group of γ-glutamyl moiety of GSH. In delta and epsilon GSTs, His-38 is maintained in most cases as a polar or charged residue and His-50 is conserved as part of an NPQHTVPTL motif. His-38 and His-50 are located within polar interaction distance of the glycyl carboxylate moiety of GSH 80, 84, 85. Ser-9 is conserved in epsilon, delta, theta, and unclassified GSTs and works to stabilize the GSH thiolate through a hydrogen bonding interaction 42, 73, 80, 83, 84, 86. In a zeta class GST of *Homo sapiens*, the GSH thiolate is stabilized by interaction with Cys-16, Ser-15, Gln-111, and Ser-14 73. In omega GSTs, BmGST-O Cys-38 is located adjacent to the GSH thiolate and dmGST-S1 Tyr-54 plays a major role in stabilizing the GSH thiolate 76, 87. The remaining amino acids that make up the core of the G-site are more variable across GSTs but are thought to aid in the positioning of GSH in the G-Site 84.

### H-site

In the GST H-site, the amino acids that contribute to the binding of multiple substrates ultimately facilitate the tolerance that an organism exhibits in a specific stress environment. Amino acid mutations in the H-site can significantly alter the catalytical activity of GST enzymes towards their substrates 88, 89. However, the sequence variability in GST active sites across species and enzyme families result in differing enzyme activities for various substrates 58. In contrast to the G-site that binds GSH across GST classes, the H-sites that bind various substrates have distinct variations in amino acid sequence and structural conformation 90. While the G-site is more hydrophilic in nature compared to the H-site, the extent of hydrophobicity of the H-site varies across GST classes and amongst individual GSTs 77, 78, 91.

In general, hydrophilic amino acids contribute to the formation of a hydrophobic pocket in the H-site adjacent to the GSH-binding site (Figure 3B&C) 80, 85. In *Anopheles gambiae*, residues in the H-site of AgGSTe2 were presumptively responsible for DDT binding and they were mostly hydrophobic residues 84. In *Plutella xylostella*, the amino acids Phe-9, Pro-10, Ile-11, Leu-14, Gly-49, Pro-52, Ala-100, and Tyr-107 are the putative H-site residues in a sigma class GST, PxGSTσ 77. Site-directed mutagenesis and inhibition assays revealed that Phe-9 is potentially an important residue for the binding of the inhibitor S-hexyl glutathione (GTX) 77. In *Blattella germanica*, Tyr-107, Tyr-115, Phe-119, and Phe-206 constitute the H-site of BgGSTD1. Purified BgGSTD1 had the highest cumene peroxidase activity among insect GSTs reported at that time that played a vital role in defending against oxidative stress 92. Studies have shown that the H-sites of different classes of GSTs are dissimilar. Diverse H-sites allow for binding and catalytic activity towards a wider range of xenobiotic substrates 93. Despite lifetime exposure to a wide variety of toxic chemicals, the presence of multiple GST classes with diverse substrate specificities facilitates an organism adaptation to adverse environments.

## Functions of insect GSTs in host plant adaptation and pesticide resistance

Many studies have found that plant allelochemicals are inducers of phase II detoxification enzymes in herbivorous arthropods 5-7. In *Choristoneura fumiferana*, the expression of *CfGST* was induced by balsam fir foliage and other multiple stresses suggesting its potential role in xenobiotic detoxification 94. The isothiocyanates produced from the breakdown of glucosinolates by the action of the enzyme myrosinase 95 are highly electrophilic, a property of a compound that makes it readily available for the nucleophilic GSH when in the presence of GST 96. Gonzalez et al. reported that the expression of *GSTD2* in *Drosophila melanogaster* was significantly higher in the taste organs (labellum and forelegs) when exposed to an isothiocyanate, insecticidal compounds naturally present in cruciferous plants 91. In addition, the mechanism of detoxification by GSTD2 was revealed via its strong affinity towards isothiocyanate and catalysis of the conjugation between GSH and isothiocyanate. Zou and others showed that glucosinolate and xanthotoxin present in *Brassica juncea* stimulated the expression of *GSTE1* in the midgut of* Spodoptera littoralis* larvae after feeding. The conjugation activity towards these allelochemicals was reduced when suppressing *GSTE1* gene expression via RNA interference (RNAi), suggesting a role for GSTE1 in host plant adaptation 97. In the Hessian fly, *Mayetiola destructor*, feeding on wheat varieties led to increased production of deterrent allelochemicals and the consequent upregulation of delta class GST genes 98. The enhanced expression of *MdesGST-1* (Delta group) in the midgut and fat body of Hessian fly larvae might explain its involvement in the detoxification of plant defense compounds such as flavonoids and scavenging endogenous ROS. Indeed, based on evidence from GST activity and RNAi studies, three GSTs are thought to have contributed to the adaptation of *N. lugens* to the host rice plant allelochemical (gramine) 99. Recently, Ma et al. identified two *Lymantria dispar* GST genes, *LdGSTe4* and* LdGSTo1* induced by host poplar allelochemicals. After silencing these two GST genes individually, the adaptation of *L. dispar* to host poplar allelochemicals was depleted 100.

Plant volatile compounds play roles in host selection by insects. For example, herbivore-induced plant volatile compounds could serve as repellents of some insects and reduce their activities, which is termed allelochemical nonpreference 4. Even for the adapted herbivore species, these volatile compounds can cause direct physiological damage to herbivores due to their neurotoxic properties at high concentrations 101, 102. As odorant degrading enzymes (ODEs), GSTs play an important role in chemoreception for the adaptation to host plant volatiles and termination of stimulation from signals (i.e., sex pheromones and plant volatiles). Antenna expressed GSTs present in the sensillar lymph of insect antennae, function in signal termination and odorant clearance, enhancing olfactory and neuron sensitivity 103-106. In *Manduca sexta*, an antenna specific GST, GST-msolf1 is expressed in the sex-pheromone-sensitive sensilla and can modify *trans*-2-hexenal, a plant derived green leaf aldehyde, suggesting its dual role in protecting sphinx moth olfactory system from harmful xenobiotics and pheromone inactivation 107. Likewise, in male silk moth (*Bombyx mori*), the antennae specific BmGSTD4 had high GSH-conjugating activity towards 1-chloro-2, 4-dinitrobenzene (CDNB), indicating its potential role in the metabolism of xenobiotics 108. Recently, the antenna expressed GmolGSTD1 was found to exhibit high degradation activity to both the sex pheromone ((Z)-8-dodecenyl alcohol) and the host plant volatile butyl hexanoate in *Grapholita molesta* 109. Most recently, the high abundance of a delta GST, *SzeaGSTd1* in *Sitophilus zeamais* antennae, inhibition of *SzeaGSTd1* catalytic activity by capryl alcohol, along with the degradation of capryl alcohol by recombinant SzeaGSTd1 were observed 110. Since capryl alcohol is a volatile component generated during grain storage, the inhibitory effects and degradation of capryl alcohol by the antenna specific SzeaGSTd1 suggest its functions in locating food and favorable oviposition site locations 110.

As phase II detoxification enzymes, arthropod GSTs confer pesticide resistance through direct metabolism or sequestration of pesticides and indirectly by providing protection against oxidative stress induced by synthetic pesticides 22. In *Rynchophorus phoenicis*, the enhanced glutathione transferase activity was associated with degradation of dichlorvos, an organophosphate insecticide 111. Yu and Killiny reported upregulation of *DcGSTe2* and *DcGSTd1* in the Asian citrus psyllid (*Di. citri*) when exposed to thiamethoxam and fenpropathrin treatment. Silencing of these GST genes enhanced mortality of Asian citrus psyllid 112. In *Tetranychus cinnabarinus*, GST *TcGSTm02* was overexpressed in a cyflumetofen resistant strain compared to a susceptible one. The activity of recombinant TcGSTm02 could be inhibited by cyflumetofen and the enzyme catalyzed the conjugation of GSH to cyflumetofen 113. Recently, RNAi-mediated knockdown of four overexpressed GST genes in the imidacloprid resistant *N. lugens* resulted in increased sensitivities to the insecticide, suggesting the roles of these GSTs in imidacloprid resistance of *N. lugens* 114. One *P. xylostella* GST, *GSTu1* upregulated in several chlorantraniliprole-resistant *P. xylostella* strains was confirmed to contribute to chlorantraniliprole resistance 115. In that study, *GSTu1* was suggested to be regulated by a novel noncoding RNA-mediated pathway 115. In *Locusta migratoria*, LmGSTE4 was found to metabolize malathion and DDT. However, insecticide bioassay showed that after suppression by RNAi, *L. migratoria* insect mortality was increased in malathion treated insects but not in deltamethrin- or DDT-treated insects 116. Most recently, 25 GST genes including 22 cytosolic and 3 microsomal genes were identified in insecticide resistance to *lambda*-cyhalothrin in *Cydia pomonella*. Among these GSTs, recombinant CpGSTd1, CpGSTd3, CpGSTe3, and CpGSTs2 could bind and metabolize *lambda*-cyhalothrin, however, no metabolites were detected. Therefore, the authors suggested that the involvement of these GSTs in *lambda*-cyhalothrin resistance might be through sequestration 117.

## Functions of insect GSTs in defense of xenobiotics induced oxidative stress

Eukaryotic cells have evolved to respond against a range of environmental stresses. Oxidative stress is a compromised state for the lipidic cell membrane due to its peroxidation by different free radicals. Pesticides produce oxidative stress in the cell, which in turn generates several ROS free radicals 50. Free radicals are atoms or molecules with unpaired electrons 118. In the quest for electronic stability, free radicals attack other molecules to stabilize their electronic state and thereby alter chemical structures and disrupt biomolecular functions 50, 118. A buildup of ROS such as H_2_O_2_ (hydrogen peroxide) and O_2_^-^ (superoxide anion) can lead to changes in metal homeostasis or oxidation states of protein metal complexes, such as the release of Fe from ferratin or the reduction of iron in cytochrome C 119. Additionally, exposure to ROS can lead to modifications that cause genomic DNA mutations, negatively affect protein activity, damage cellular membranes, and eventually leading to cell death. Evolutionarily, GSH has been one of the key nucleophilic chemicals in living organisms that convert a range of electrophilic compounds into a less toxic form 120, 121. In the case of redox stress, two molecules of GSH reduce one molecule of hydrogen peroxide in the presence of glutathione peroxidases, generating one molecule of glutathione disulfide (GSSG), an oxidized form of GSH, and two molecules of water 122, 123. The glutathione peroxidase, which is responsible for protecting lipids and proteins from oxidation, is regulated by the essential trace metal element Selenium (Se) 124. The Se-dependent glutathione peroxidase metabolizes hydrogen peroxides and hydroperoxides 40, 125. In the absence of Se, GST performs glutathione peroxidase activity mostly towards organic hydroperoxides 121, 126, 127. Once GSSG is formed, flavin adenine dinucleotide (FAD)-dependent enzyme glutathione reductase transfers electrons from NADPH, regenerating two molecules of GSH 121.

Many Se-independent peroxidase reactions performed by GSTs in insects have been reported. In *Dr. melanogaster,* DmGSTS1-1 exhibited glutathione peroxidase activity towards cumene hydroperoxide (CHP, oxidative stress inducer). Since *DmGSTS1-1* was highly expressed in the flight muscle, the localization of the corresponding GST enzyme might provide a protective role against oxidative stress generated from mitochondrial respiration 128. Similarly, Sawicki and others found six delta class GST genes (*GSTD1*, *GSTD2*, *GSTD3*, *GSTD7*, *GSTD9*, and *GSTD10*), one epsilon class GST (*GSTE1*), and one sigma class GST gene (*GSTS1*) in *Dr. melanogaster* that could conjugate 4-hydroxynonenal (4-HNE), an electrophilic end-product of lipid peroxidation 129. The role of GSTs in attenuating pyrethroid-induced oxidative stress, which conferred insecticide resistance in the rice brown planthopper (*N. lugens*) was highlighted by Vontas et al. 46. It was reported that the increase in GST-based peroxidase activity and the increased amount of GSH indicated the role of GST in reducing the damage from pesticide-induced oxidative stress. Zhang and others showed GSTO2 in Apis cerana cerana had peroxidase activity toward CHP and t-butylhydroperoxide 130. Similarly, a defensive role against oxidative stress by RpGSTO1 towards different concentrations of CHP was observed in the bird cherry-oat aphid, *Rhopalosiphum padi* 131. The GST antioxidant role has also been highlighted in an urban pest, the German cockroach *B. germanica*. Cockroaches exhibited high GSTD1 peroxidase activity against CHP, indicating a role in insecticide metabolism and reduction of redox stress 92. Similarly, GSTE1-1 in both DDT resistance and susceptible *An*. *gambia*, showed peroxidase activity with CHP but was unable to perform dehydrochlorination activity. The opposite result was obtained for GSTE2-2, indicating these two GSTs play an important role in gaining resistance to DDT via conjugation and peroxidase activity, respectively 49. In two-week-old adults of Ap. cerana cerana, the expression of *AccGSTS1* was high when exposed to various environmental stressors such as temperature (cold and heat shock), heavy metal (HgCl_2_), pesticides (phoxim, cyhalothrin, and acaricide), H_2_O_2_, and ultraviolet 45 radiation which are known for their property to generate oxidative stress 55. The researchers observed dose-dependent removal of H_2_O_2,_ indicating *AccGSTS1* functions in the elimination of oxidative stress 55. A similar result was obtained for *AccGSTZ1* in Ap. cerana cerana when exposed to varying temperatures and H_2_O_2,_ suggesting a protective function against oxidative stress 132.

During evolution, insects have adapted to stresses posed by plant-derived toxic chemicals. When feeding on plant species in the Apiaceae or Rutaceae families, which contain furanocoumarin- a toxic photoactive pro-oxidant, *Papilio polyxenes* exhibited significantly higher GST-mediated peroxidase activity. This is indicative of an insect adaptive mechanism against oxidative stress generated by the plant-derived toxic chemical substances 54, 133, suggesting many GSTs are responsible for protecting tissues and reducing the mortality rate of insects caused by oxidative stress. There are also some cases where specific GSTs are not able to conduct peroxidase activity, such as theta class GSTs 134, 135. Interestingly, some insects do not have Se-dependent glutathione peroxidases or have enzymes with limited expression and/or activity 49, 54, 127, 136. Finding evidence on how insects eliminate oxidative stress in the absence of Se-dependent glutathione peroxidase for survival or adaptations to environmental stressors is the direction of future research.

## Conclusions

GSTs play a vital role in detoxifying or metabolizing a diverse range of chemical compounds, of xenobiotic or endobiotic origin. GST mediated detoxification is critical for adaptation against xenobiotics including plant allelochemicals and synthesized pesticides. GSTs confer adaptation to a diverse range of xenobiotics through metabolism or sequestration of chemicals and protection against chemical induced oxidative stress. The key to the diverse roles of different classes of GSTs is due to their structure, specifically the composition and spatial localization of amino acid residues composed in the enzymatic active sites. Through a combination of arthropod structural biology, enzyme kinetics and site-directed mutagenesis techniques, our understanding of such diversity in GST structural and functional complexity can be improved.

## Figures and Tables

**Table 1:** GST gene number in diverse species across six insect orders

Order	Name	Type	Delta	Epsilon	Omega	Sigma	Theta	Zeta	Unclassified	Microsomal	Total	Reference
Coleoptera	*Leptinotarsa decemlineata*	Pest (Oligophagous)	3	10	5	4	4	1	2	1	30	66
	*Tribolium castenaum*	Pest (Polyphagous)	3	19	3	7	1	1	2	5	41	64
Diptera	*Aedes aegypti*	Pest (Oligophagous)	8	8	1	1	4	1	3	-	26	138
	*Anopheles gambiae*	Pest (Sanguivorous, Oligophagous)	12	8	1	1	2	1	3	3	31	72
	*Bactrocera dorsalis*	Pest (Polyphagous)	4	8	2	0	1	1	1	-	17	139
	*Chironomus riparius*	Pest (Sanguivorous, Oligophagous)	3	1	1	4	1	1	2	-	13	140
	*Culex quinquefasciatus*	Pest (Sanguivorous, Oligophagous)	14	9	1	1	6	0	4	5	40	141
	*Drosophila melanogaster*	Pest (Polyphagous)	11	14	5	1	4	2	0	1	38	72, 149
Hemiptera	*Bemisia tabaci*	Pest (Polyphagous)	14	0	1	6	0	2	-	2	25	142
	*Diaphorina citri*	Pest (Oligophagous)	2	2	0	3	0	0	1	2	11	142
	*Myzus persicae*	Pest (Polyphagous)	8	0	0	8	2	0	0	2	21	143
	*Nilaparvata lugens*	Pest (Monophagous)	2	1	1	3	1	1	0	2	11	144
	*Orius laevigatus*	Predator (Polyphagous)	1	0	2	16	1	1	0	3	24	145
Homoptera	*Acyrthosiphon pisum*	Pest (Oligophagous)	10	0	2	6	2	0	0	2	22	144
Hymenoptera	*Apis mellifera*	Pollinator (polyphagous)	1	0	1	4	1	1	0	2	10	72
	*Nasonia vitripennis*	Parasitoid (Monophagous)	5	0	2	8	3	1	0	-	19	146
Lepidoptera	*Bombyx mori*	Economic (Monophagous)	4	8	4	2	1	2	2	-	23	147
	*Plutella xylostella*	Pest (Oligophagous)	5	5	5	2	1	2	2	-	22	65
	*Spodoptera litura*	Pest (Polyphagous)	5	21	3	7	1	5	3	2	47	148

-: There is no known gene in these classes.

## Abbreviations

GSTs: glutathione S-transferases
PIs: protease inhibitors
HIPVs: herbivore-induced plant volatiles
P450s: cytochrome P450 monooxygenases
COEs: carboxylesterases
ABC transporters: ATP-binding cassette transporters
UGTs: UDP-glycosyltransferases
GSH: glutathione
ROS: reactive oxygen species
RNAi: RNA interference
ODEs: odorant degrading enzymes
GSSG: glutathione disulfide
CHP: cumene hydroperoxide
4-HNE: 4-hydroxynonenal
CDNB: 1-chloro-2, 4-dinitrobenzene
